# Macrophage migration inhibitory factor alleviates irradiation induced apoptosis of BMSCs through the LncRNA MEG3/NOX4 axis

**DOI:** 10.1515/med-2025-1339

**Published:** 2026-02-13

**Authors:** Kai Hu, Han Zhou, Hongwei Hu, Zhibiao Bai, Lingwei Mou, Shaohao Wu, Zeyu Shou, Changbao Liu, Chun Chen

**Affiliations:** The Dingli Clinical College of Wenzhou Medical University, Wenzhou, People’s Republic of China; Department of Orthopaedics, The First Affiliated Hospital of Wenzhou Medical University, Wenzhou 325000, People’s Republic of China; Zhejiang Engineering Research Center for Hospital Emergency and Process Digitization, Wenzhou 325000, Zhejiang, People’s Republic of China; The Second Affiliated Hospital and Yuying Children’s Hospital of Wenzhou Medical University, Wenzhou, Zhejiang, People’s Republic of China; Key Laboratory of Intelligent Treatment and Life Support for Critical Diseases of Zhejiang Province, Wenzhou 325000, Zhejiang, People’s Republic of China

**Keywords:** macrophage migration inhibitory factor, LncRNA MEG3, BMSCs, NOX4, irradiation

## Abstract

**Objectives:**

This study aimed to explore the mechanism by which exogenous macrophage migration inhibitory factor (MIF) reduces apoptosis of mouse bone marrow mesenchymal stem cells (BMSCs) induced by irradiation under high oxidative stress.

**Methods:**

BMSCs were cultured and exposed to irradiation using a linear accelerator to establish a radiation damage model. Cell viability was detected by the CCK-8 assay. Apoptosis rate and intracellular reactive oxygen species (ROS) production were measured by flow cytometry and fluorescence microscopy. The expression levels of Long non-coding RNA (lncRNA) MEG3, NOX4, and apoptosis-related genes were detected by western blot, real-time quantitative polymerase chain reaction (RT-qPCR), and immunofluorescence.

**Results:**

The CCK-8 assay, western blot, and flow cytometry confirmed that irradiation effectively induced BMSCs apoptosis and upregulated the expression of lncRNA MEG3, which was positively correlated with NOX4 expression. Western blot, immunofluorescence, and RT-qPCR results demonstrated that MIF protected BMSCs from irradiation-induced apoptosis and downregulated the expression of MEG3 and NOX4. Furthermore, MEG3 siRNA was shown to reduce irradiation-induced apoptosis. Western blot and flow cytometry analyses revealed that overexpression of either NOX4 or MEG3 could reverse the protective effect of MIF.

**Conclusions:**

LncRNA MEG3 is an important regulatory factor in irradiation-induced apoptosis. The mechanism by which MIF protects BMSCs from irradiation-induced apoptosis is likely mediated through the regulation of the LncRNA MEG3/NOX4 signaling pathway.

## Introduction

Radiotherapy-induced avascular necrosis of the femoral head (RANFH) is one of the major complications associated with clinical radiotherapy and accidental radiation exposure [1]. Studies have reported a 17 % incidence of necrotic fractures in patients with uterine cancer who received radiation therapy [2]. While conventional surgical interventions remain a primary treatment option, alternative therapeutic approaches such as autologous stem cell implants and hyperbaric oxygen therapy have gained popularity in recent years [3], 4]. Among these, mesenchymal stem cell (MSC) therapy has been widely adopted in clinical practice due to its proven safety profile [5]. However, there remain some problems such as low survival rate of transplanted cells limiting its therapeutic efficacy [6]. For example, oxidative stress exists in the microenvironment of most lesions, which limits the survival rate and cell bioactivity of transplanted BMSCs, affecting the curative effect [7]. With the development of bone tissue engineering, genetic engineering is the key of RANFH tissue regeneration therapy [8]. Consequently, there is a pressing need to explore the underlying mechanisms of RANFH to enhance the antioxidant stress capacity of transplanted BMSCs, ultimately improving their survival and therapeutic potential.

LncRNA plays an important role in osteonecrosis of the femoral head [9]. Defined as endogenous RNA strands longer than 200 nucleotides that do not encode proteins, lncRNAs modulate cellular processes such as energy metabolism and life activities through interactions with proteins and miRNAs [10], 11]. Previous studies have shown that lncRNA participates in the regulation of cell proliferation, autophagy, apoptosis, migration and other important biological processes by regulating the downstream signal pathways [12], 13]. Among these, lncRNA MEG3, the first identified tumor-suppressing lncRNA, plays a pivotal role in BMSC proliferation and apoptosis [14]. For example, lncRNA MEG3 has been shown to mitigate inflammation, oxidative stress, and apoptosis in Cajal interstitial cells induced by TNF-α [15]. Additionally, it inhibits synovial cell proliferation and promotes apoptosis in rat models of knee osteoarthritis by regulating PTEN [16]. Conversely, lncRNA MEG3 exacerbates apoptosis in renal tubular epithelial cells under hypoxia/reoxygenation through the miR-129-5p/HMGB1 axis [17]. Modulating lncRNA MEG3 expression may thus represent a promising strategy to enhance the survival of transplanted BMSCs. Despite these findings, the role of MEG3 in BMSCs apoptosis within RANFH remains unexplored.

MIF is an evolutionarily conserved protein, which was first identified as a molecule secreted by activated T lymphocytes more than 50 years ago to inhibit macrophage migration in capillaries [18], has recently been implicated in apoptosis regulation. In BMSCs subjected to ischemia and hypoxia, MIF expression is significantly reduced, correlating with increased apoptosis, while exogenous MIF supplementation markedly attenuates this effect [19]. Furthermore, research also indicates that MIF can promote the growth of epithelial cells of benign prostatic hyperplasia by regulating COX-2 and p53 signal transduction pathway [20]. The above related experiments clearly suggest that MIF is closely related to apoptosis and growth. Regulating the expression of MIF may be one of the effective ways to interfere with the apoptosis of transplanted cells. For instance, in smoking-induced emphysema models, MIF inhibits the p53 signaling pathway, reducing apoptosis and DNA damage in pulmonary epithelial cells [21]. Similarly, MIF has been shown to suppress p53 transcription in brain tumors, thereby inducing tumor cell apoptosis [22]. These findings suggest MIF’s dual role in apoptosis regulation, yet its therapeutic potential in RANFH remains unclear.

MIF is an evolutionarily conserved protein, first identified over 50 years ago as a molecule secreted by activated T lymphocytes that inhibits macrophage migration [18]. Beyond its role in immunity, MIF has garnered attention for its involvement in apoptosis regulation. In BMSCs under ischemia and hypoxia, MIF expression is significantly reduced, correlating with increased apoptosis, while exogenous MIF supplementation markedly attenuates this effect [19]. Additionally, MIF promotes the growth of benign prostatic hyperplasia epithelial cells via regulation of the COX-2 and p53 signaling pathways [20]. These studies underscore MIF’s relevance in both apoptosis and cell growth, suggesting that modulating MIF expression could be a viable strategy to influence transplanted cell survival. Notably, MIF also engages in redox regulation and interacts with noncoding RNAs, further expanding its functional repertoire. For instance, MIF exhibits tautomerase activity and can modulate reactive oxygen species (ROS) production through interactions with pathways involving antioxidant enzymes and redox-sensitive transcription factors (e.g., NF-κB) [23].though specific mechanisms in stem cells remain to be fully elucidated. Moreover, MIF has been shown to influence the expression of various noncoding RNAs in different pathological contexts, which may contribute to its regulatory effects on apoptosis and proliferation. For example, in smoking-induced emphysema models, MIF inhibits the p53 pathway, reducing DNA damage and apoptosis in lung epithelial cells [21]. Conversely, MIF suppression promotes p53 transcription and apoptosis in brain tumors [22], highlighting its context-dependent roles. Despite these advances, MIF’s therapeutic potential in RANFH, particularly through redox or ncRNA-related mechanisms, remains unclear.

Recent studies reveal that MIF interacts with lncRNAs to regulate apoptosis. For instance, MIF protects MSCs from ischemia/hypoxia-induced apoptosis via lncRNA p21 [24]. Given that lncRNA MEG3 is a key effector of the p53 signaling pathway [25], it is plausible that MIF may regulate lncRNA MEG3 to influence cellular apoptosis. For example, overexpression of lncRNA MEG3 has been shown to promote p53 expression and inhibit cell proliferation [26]. Similarly, the chemotherapeutic agent Fenofibrate enhances lncRNA MEG3 expression, activating the p53 pathway to suppress tumorigenesis [27]. Moreover, Post-radiotherapy ischemia/hypoxia could upregulate MEG3, promoting apoptosis in transplanted BMSCs, whereas exogenous MIF counteracts this effect by suppressing p53-mediated MEG3 expression, thereby improving therapeutic outcomes in RANFH.

NADPH oxidase 4 (NOX4) is recognized as a critical regulator of cell differentiation, proliferation and apoptosis [28]. Particularly in stem cells, NOX4 activation has been linked to increased apoptosis, highlighting its pivotal role in tissue regeneration and cell fate determination [29]. Meanwhile, macrophage migration inhibitory factor (MIF) has been identified as a key upstream activator of the NOX4 pathway [30]. Notably, beyond protein-level regulation, emerging evidence suggests important roles for long non-coding RNAs (lncRNAs) in oxidative stress pathways. A seminal study demonstrated a direct regulatory axis where lncRNA MEG3 modulates NOX4 expression in endothelial cells [31], suggesting potential cross-talk between lncRNAs and oxidative stress regulation. However, despite these advances, the role of such regulatory mechanisms in BMSCs remains largely unexplored. Specifically, whether the MEG3-NOX4 regulatory axis exists in BMSCs, and more importantly, whether it governs cellular fate decisions through modulation of oxidative stress-induced apoptosis, are completely unknown. Based on this scientific premise, we hypothesized that in BMSCs, MEG3 acts as an upstream regulator of NOX4 to modulate oxidative stress-induced apoptotic pathways. This study aims to systematically investigate this novel MEG3-NOX4 signaling axis in BMSCs, which may provide new insights into the regulation of stem cell fate under oxidative stress conditions.

In this study, we propose that MIF attenuates apoptosis in transplanted BMSCs by downregulating lncRNA MEG3, thereby inhibiting NOX4-mediated oxidative stress. This mechanism could enhance the survival and therapeutic efficacy of stem cell transplantation in RANFH. Further investigation into the MIF-MEG3-NOX4 axis may provide novel targets for improving regenerative therapies in radiation-induced osteonecrosis.

## Materials and methods

### Isolation of BMSCs

BMSCs were harvested from the bone marrow of C57BL/6 mice. Briefly, bone marrow cells were flushed out and collected from the femur and tibia of mice, plated in 60 mm cell culture dishes, and cultured overnight in a 37 °C incubator with 5 % CO_2_. Then, nonadherent cells were removed by rinsing twice with phosphate-buffered saline (PBS). The adherent cells were maintained in Dulbecco’s Modified Eagle Media (DMEM) containing 10 % fetal bovine serum (FBS), 2 mM Glutamax, and 1 % penicillin and streptomycin (PS).

### Cell culture

The BMSCs were plated in cell culture plates (60 mm diameter) in DMEM-F12 medium (Gibco, USA) with 10 % fetal bovine serum (FBS) (EverGreen, China). Cells were maintained in a humidified incubator with an atmosphere of 37 °C and 5 % CO_2_. The medium was replaced every 2–3 days. The cells were passaged with 0.25 % trypsin after reaching 90 % confluence.

### Irradiation

A Synergy VMAT linear accelerator was purchased from ELEKTA (Stockholm, Sweden). BMSCs were plated in 60-mm culture dishes and a dose rate of 1.02 Gy/min was used to establish the radiation damage models [32].

### Cell treatment

In this study, 10Gy radiation dose pretreatment was used to establish the cell apoptosis model to explore the role of MIF, lncRNA MEG3 and NOX4 in the pathological process of radiation-induced apoptosis of BMSCs and their interaction relationship. Then, the anti-radiation effect of MIF was detected by exposing BMSCs to the radiation with or without pretreatment of MIF (MCE, USA) for 24 h. Plasmids containing lncRNA MEG3, NOX4 and siRNA MEG3 were used to overexpress or silence the corresponding gene, and vector or NC siRNA was used as a negative control. Cells and cultural supernatants were collected for the subsequent experiments of western blot or qRT-PCR.

### Cell viability assay

The viability of BMSCs was evaluated by Cell Counting Kit-8 (CCK-8) (Beyotime, China) analysis in line with the manufacturer’s instructions. In brief, 1×10^4^ cells were seeded into each well of a 96-well plate for 24 h. When cell growth to 85–90 % confluence degree, they were exposed to IR or drugs at different concentrations for a certain period. Then, 10 µL of CCK-8 reagent was added to each well and co-incubated for 2 h at 37 °C. Eventually, with the aid of a microplate reader (Molecular Devices, SPECTRA max Plus, USA), OD value at 450 nm was measured.

### Total RNA extraction and quantitative real-time polymerase chain reaction (RT-qPCR) analysis

Total mRNA was isolated according to the manufacturer’s protocol by using RNA Extraction Kit with Spin Column (Beyotime, China). In a 20 µL reaction system, a cDNA Kit (TOROIVD, China) was used to synthesize the cDNA. qRT-PCR was carried out in QuantStudio5 real-time PCR system (ThermoFisher, USA). The PCR cycles were set at 95 °C for 60 s, and then 40 cycles at 95 °C for 10 s and annealing/extension at 60 °C for 30 s. The related mRNA expression was determined using the 2^−ΔΔCt^ method. All forward and reverse primers used were synthesized by Sangon Biotech (Shanghai, China). β‐actin were used as the internal control. The primer sequences used for amplification were shown in Table 1.

**Table 1:** Primer sequences utilized for RT-PCR analyses.

Gene	Sequence
NOX4	

Forward	5′-TCA​TGG​ATC​TTT​GCC​TCG AGGGTT-3′
Reverse	5′-AGTGACTCCTCAAATGGGCTTCCA-3′

MEG3	

Forward	5′-GTGGACAATGGTGTCCAGGC-3
Reverse	5′-TTAACTCAGAGCGGGTCTCC-3′

β-actin	

Forward	5′-GGA​CTC​GTC​ATA​CTC​CTG​CTT​G-3′
Reverse	5′-GGA​AAT​CGT​GCG​TGA​CAT​TAA​G-3′

### Protein isolation and western blotting

According to the manufacturer’s instructions, 200 μL RIPA Lysis Buffer (Beyotime, China) containing 1 % PMSF was used to extract total protein from cells at 4 °C for 15–20 min after PBS washing. Then collected liquid and centrifuged at 12,000 g for 10 min at 4 °C. After centrifugation, the concentration of the protein in the supernatant was measured by the bicinchoninic acid (BCA) method. After loading buffer was added, proteins (30 μg) from each sample were electrophoresed on 12.5 % SDS-PAGE gels, transferred to PVDF membranes, and blocked with Blocking Buffer(Beyotime, China) for 12 min at room temperature. Subsequently, the membranes were incubated with the following primary antibodies at 4 °C overnight: anti-Bax (1/5,000–1/12,000, Proteintech Group, Inc, USA, Cat. No.: 505599-2-Ig), anti-Bcl2 (1/1,000-1:4,000, Proteintech Group, Inc, USA, Cat. No.: 25593-1-1-AP), anti-caspase3 (1/500-1:2000, Proteintech Group, Inc, USA, Cat. No.: 19677-1-AP), anti-NOX4 (1/500–1/2000, Proteintech Group, Inc, USA, Cat. No.: 14347-1-AP), and anti-β-actin (1/3,000–1/20,000, Affinity, USA, Cat. No.: AF7018). After washing with TBST three times, the membranes were treated with the corresponding horseradish peroxidase (HRP)-conjugated secondary antibody for 1 h and incubated at room temperature, as well as visualizing the expression of immunoreactive protein bands by the chemiluminescence imaging system (Bio-Rad, USA) and quantified using Image J.

### Apoptotic incidence detection

We applied flow cytometry to detect the apoptosis of BMSCs after treatment. BMSCs were collected and washed with PBS and then resuspended in 100 µL staining buffer with 5 µL Annexin V and 10 µL PI dye (YEASEN, Shanghai, CHINA) at 37 °C for 30 min. The apoptosis rate was assessed by flow cytometry (BD Biosciences, USA) within 1 h. Then the results were analyzed by CytExpert software (BD Biosciences, USA).

### Hoechst 33,342 fluorescence staining

Apoptotic cells were detected by use Hoechst 33,342 staining solution ((Beyotime, China) following the manufacturer’s protocol. Briefly, the cells were washed twice with PBS and incubated with Hoechst 33,342 at 4 °C for 30–40 min. Subsequently, the cells were washed three times with PBS and the blue fluorescence emitted by the cells was observed under a fluorescence microscope (Leica, Germany). The living cells were bright blue, and the fluorescence intensity of apoptotic cells was significantly strengthened.

### Immunostaining

Immunofluorescent staining of NOX4 was performed according to typical protocols. Shortly, the treated cells in 24-well plates were washed twice with PBS for 5 min, the glass slides in the 24-well plates were removed, then fixed with 4 % paraformaldehyde at room temperature for 30 min. Cells were permeabilized with 0.1 % Triton X-100 (Beyotime, China) for 15 min, then blocked with 1 % BSA (Beyotime, China) for 30 min. Cells were then incubated with 1 % BSA diluted primary antibody against NOX4 (1:25 dilution) (Proteintech Group, Inc, USA, Cat. No.: 14347-1-AP) at 4 °C refrigerators overnight, following incubation with the secondary antibody of the corresponding species at room temperature for 1 h. The nuclei of BMSCs were stained with 5ug/mL DAPI (Beyotime, China). After each step, the cells were washed with PBS buffer for 3 times. Finally, drops of anti-fluorescence quench agent were added and covered with a cover slide. Finally, fluorescence microscope (Leica, Germany) was used to record fluorescence images.

### ROS detection

BMSCs were inoculated at 1×10^5^ cells/well and cultured for 24 h. The treated cells were collected and resuspended with a solution of the ROS-indicator dye DCFH-DA (10 μM) (Beyotime, China) in the serum-free medium for 20 min at 37 °C under 5 % CO_2_ and avoided light in the whole process. Entirely suspension the solution every 3–5 min. After 20 min, the dye-containing medium was eliminated and the cells were washed in triplicate with the serum-free medium to remove the residual DCFH-DA. After rinsing, fluorescent signals were immediately measured by flow cytometry or observed by fluorescence microscopy.

### Statistical analysis

All numerical data were expressed as mean±standard deviation (SD) and analyzed by SPSS 22 (IBM, USA). Differences between the two groups were analyzed using Student’s t-test. The histograms were made with GraphPad9.0(GraphPad Software Inc., CA). Differences were considered to be significant when p<0.05. The *, ** and *** in figures indicated p values<0.05, 0.01, and 0.001 respectively.

## Results

### MIF could protect BMSCs from apoptosis induced by IR

**Figure 1:**
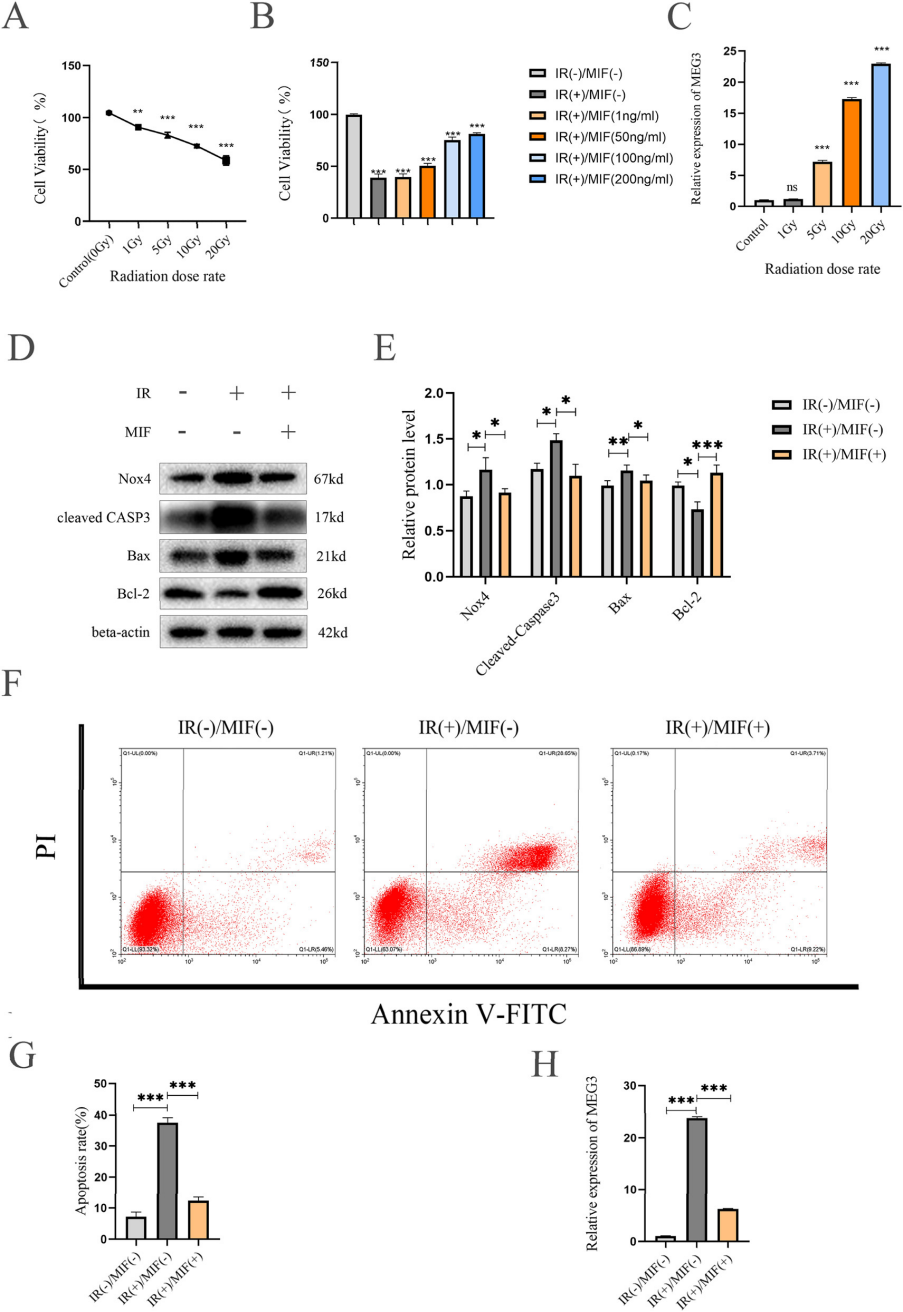
MIF protects BMSCs against apoptosis induced by IR. (A) BMSCs irradiated with different doses (0 Gy,1 Gy,5 Gy,10 Gy,20 Gy) X-ray, and cultured for another 24 h after receiving radiation. The cell viability of BMSCs was detected by CCK-8 assay. (B) The results of CCK-8 detection of BMSCs exposed to 10 Gy X-ray after treatment with MIF (0,1,50,100, and 200 ng/mL) for 24 h. (C) The lncRNA MEG3 level of BMSCs treated with different doses (0 Gy,1 Gy,5 Gy,10 Gy,20 Gy) X-ray (D) western blot was used to detect the expression of NOX4 and apoptosis-related proteins in BMSCs of different groups. (E) The quantification of the data in panel D. (F) Annexin V/PI double staining and flow cytometry were used to detect the percentage of apoptosis of BMSCs in control group and IR treated group and MIF treated group before IR treatment. (F) Quantification of the percentage of apoptotic BMSCs, as shown in panel E. (G) RT-qPCR detected the expression of lncRNA MEG3 in IR-treated group, non-IR-treated group, and MIF-treated group before IR treatment. (n=3, *p<0.05, **p<0.01, ***p<0.001 using the t-test).

Current studies have explored whether MIF can protect BMSCs and its mechanisms. CCK-8 test results showed that when the radiation dose was 10Gy and 20Gy, the cell viability decreased about 24 % (***p<0.001) and 40 % (***p<0.001) compared with the control group (Figure 1A). At the same time, the cell activity increased as the concentration of pretreated MIF increases, and the results showed that MIF exhibited a significantly protective effect when the concentration of MIF was more than 100 ng/mL (Figure 1B). RT-qPCR analysis revealed that lncRNA MEG3 expression in BMSCs was significantly upregulated by approximately 17-fold (***p<0.001) and 23-fold (***p<0.001) after exposure to 10 Gy and 20 Gy irradiation, respectively, compared with the control group. (Figure 1C). Western blot analysis revealed that pretreatment with 100 ng/mL MIF for 24 h significantly modulated the expression of apoptosis-related proteins and Nox4 in BMSCs following 10 Gy IR treatment. Compared to IR-treated BMSCs without MIF pretreatment, the pro-apoptotic proteins Bax and cleaved caspase-3 were downregulated by 10 % (*p<0.05) and 26 % (*p<0.05), respectively, while the anti-apoptotic protein Bcl-2 was upregulated by 54 % (***p<0.001). Concurrently, Nox4 expression was downregulated by 21 % (*p<0.05) (Figure 1D and E). Flow cytometric analysis showed that MIF pretreatment reduced the proportion of apoptotic cells from 36.9 to 12.9 % (*p<0.001), representing a 65 % decrease (Figure 1F and G). In addition, RT-qPCR detection showed that IR treatment could significantly upregulate the expression of lncRNA MEG3, which was downregulated by pretreatment of MIF (Figure 1H).

### Overexpression of LncRNA MEG3 reversed the protective effect of MIF on BMSCs and activated NOX4

**Figure 2:**
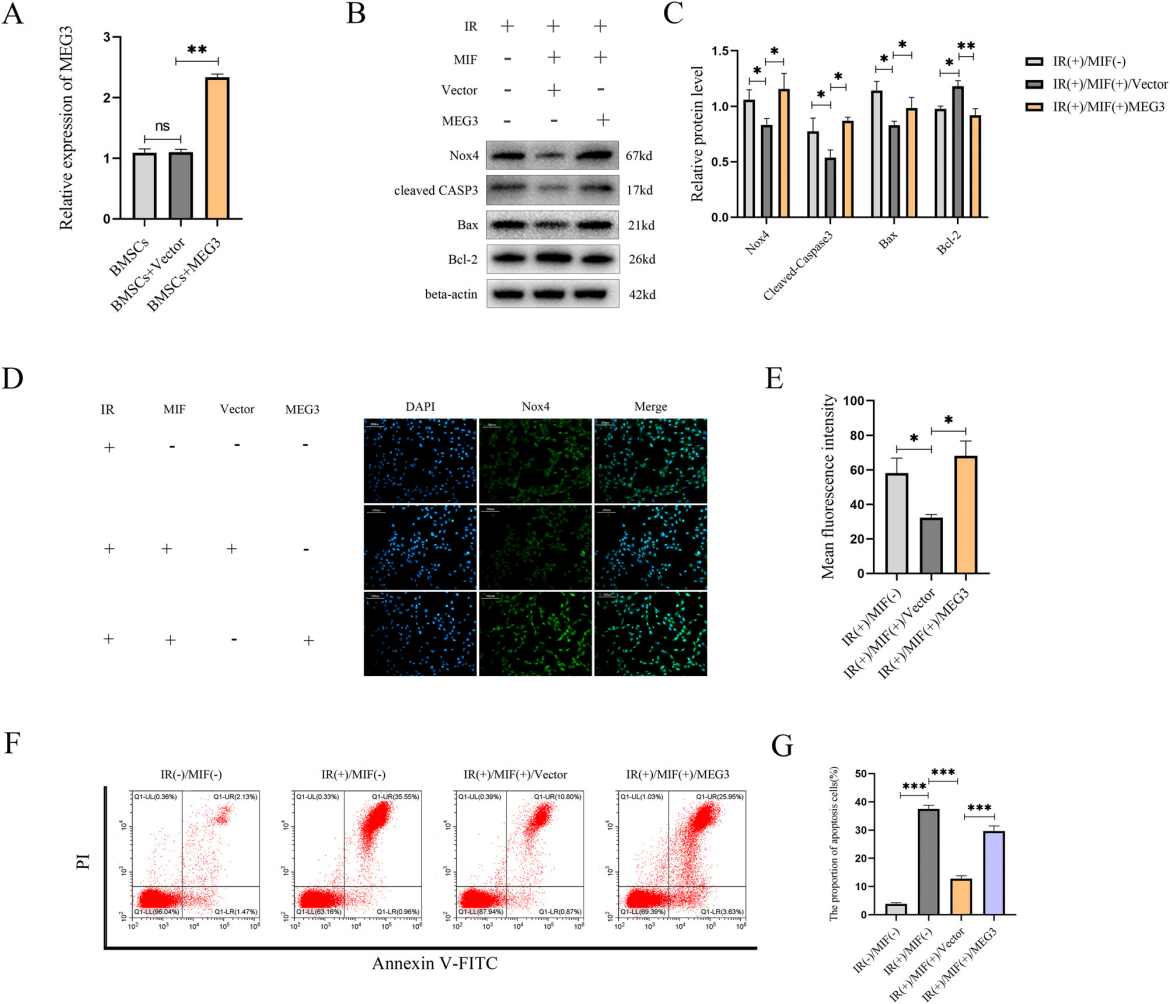
Overexpression of lncRNA MEG3 weakened the cytoprotective effect of MIF and activated NOX4. (A) The mRNA levels of MEG3 of BMSCs pretreated with or without lncRNA MEG3 plasmid or non-targeted mRNA (vector) before treatment of MIF and IR were detected by RT-qPCR. (B) The expression of NOX4 and apoptosis-related proteins of BMSCs pretreated with or without MIF or plasmid containing lncRNA MEG3 or non-targeted mRNA (vector) before treatment of IR were detected by western blot. (C) Quantification of data in panel B. (D) The effect of overexpression of lncRNA MEG3 on the expression of NOX4 in BMSCs pretreated with MIF and treated with IR was analyzed by immunofluorescence. The nucleus and NOX4 were stained with blue and green fluorescent dyes, respectively. (E) The quantification of data in panel D. (F) Annexin V/PI double staining and flow cytometry were used to analyze the apoptosis of cells transfected with lncRNA MEG3 plasmid or vector without MIF or combined with MIF pretreatment. (G) Quantification of the proportion of apoptotic BMSCs detected in panel F. (n=3, no significance was noted as “ns”, *p<0.05, **p<0.01, ***p<0.001 using the t-test).

To investigate the effect of lncRNA MEG3 on IR-induced apoptosis in BMSCs, BMSCs were transfected with an overexpressed lncRNA MEG3 plasmid or empty plasmid, pretreated with MIF for 24 h, and then treated with IR. Transfection with the lncRNA MEG3-expressing plasmid significantly upregulated lncRNA MEG3 mRNA expression by 2.3-fold (**p<0.01). compared to the control group. (Figure 2A). Western blot analysis revealed that transfection of BMSCs with the lncRNA MEG3 overexpression plasmid significantly modulated the expression of apoptosis-related proteins and Nox4. Compared to the Vector group, the pro-apoptotic proteins Bax and cleaved caspase-3 were upregulated by 24 % (*p<0.05) and 78 % (*p<0.05), respectively, while the anti-apoptotic protein Bcl-2 was downregulated by 22 % (**p<0.01). Concurrently, Nox4 expression was upregulated by 38 % (*p<0.05). Immunofluorescence and western blot results showed that MIF pretreatment significantly downregulated the expression of NOX4 in BMSCs treated with IR. However, when the lncRNA MEG3 was overexpressed, the expression of NOX4 was upregulated (Figure 2B–E). Transfection of BMSCs with the lncRNA MEG3 overexpression plasmid significantly increased the proportion of apoptotic cells (29.58 %) compared to the Vector group (11.67 %; ***p<0.001) (Figure 2F and G). These data suggest that overexpression of lncRNA MEG3 can reverse the protective effect of MIF on BMSCs from IR-induced apoptosis and upregulate NOX4 expression.

### Downregulation of lncRNA MEG3 plays a protective role in BMSCs and inhibits NOX4

**Figure 3:**
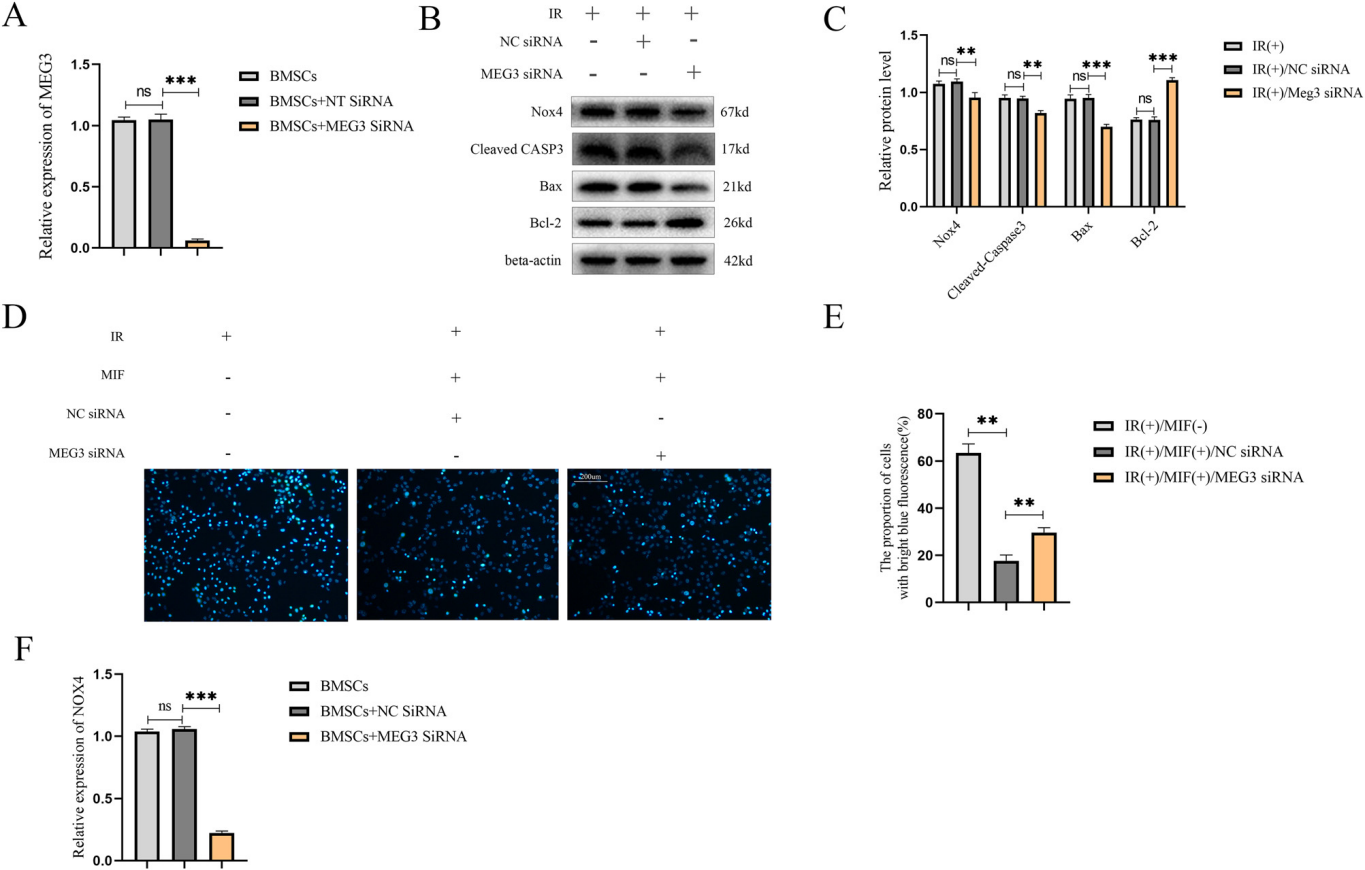
Downregulation lncRNA MEG3 protects BMSCs and inhibits NOX4. (A) RT-qPCR is used to verify the efficiency of lncRNA MEG3 siRNA. (B) The expression of NOX4 and apoptosis-related proteins in BMSCs transfected with lncRNA MEG3 siRNA or NC siRNA before IR treatment was detected by western blot. (C) The quantification of data in panel B. (D) Hoechst33342 staining showed the percentage of apoptotic cells with bright blue fluorescence in BMSCs transfected with lncRNA MEG3 siRNA under IR treatment was significantly lower than that under IR treatment with NC siRNA transfection. (E) The quantification of data in panel D. (F) The mRNA levels of NOX4 of BMSCs pretreated with or without MEG3 siRNA or NC siRNA before treatment of MIF and IR were detected by RT-qPCR. (n=3, no significance was noted as “ns”, *p<0.05, **p<0.01, ***p<0.001 using the t-test).

To further verify the effect of lncRNA MEG3 on IR-induced apoptosis in BMSCs, the levels of NOX4 and apoptosis-related proteins were measured after IR treatment. Compared with the NC siRNA group, the expression of pro-apoptotic proteins was significantly downregulated in the MEG3 siRNA group, with Bax reduced by 26 % (***p<0.001) and cleaved caspase-3 decreased by 14 % (**p<0.01). In contrast, the anti-apoptotic protein Bcl-2 was upregulated by 44 % (***p<0.001). Concurrently, NOX4 expression was downregulated by 12 % (**p<0.01) following MEG3 knockdown. (Figure 3B and C). Hoechst33342 staining showed that the proportion of apoptotic cells in the MEG3 siRNA group (30 %) was significantly lower than that in the NC siRNA group (17 %) (**p<0.01) (Figure 3D and E). The above data suggest that knockdown of lncRNA MEG3 can protect BMSCs from IR-induced apoptosis. At the same time, RT-qPCR analysis showed that knockdown of lncRNA MEG3 could significantly downregulate the expression of the NOX4 gene (Figure 3F).

### Overexpression of NOX4 reversed the protective effect of MIF on BMSCs

**Figure 4:**
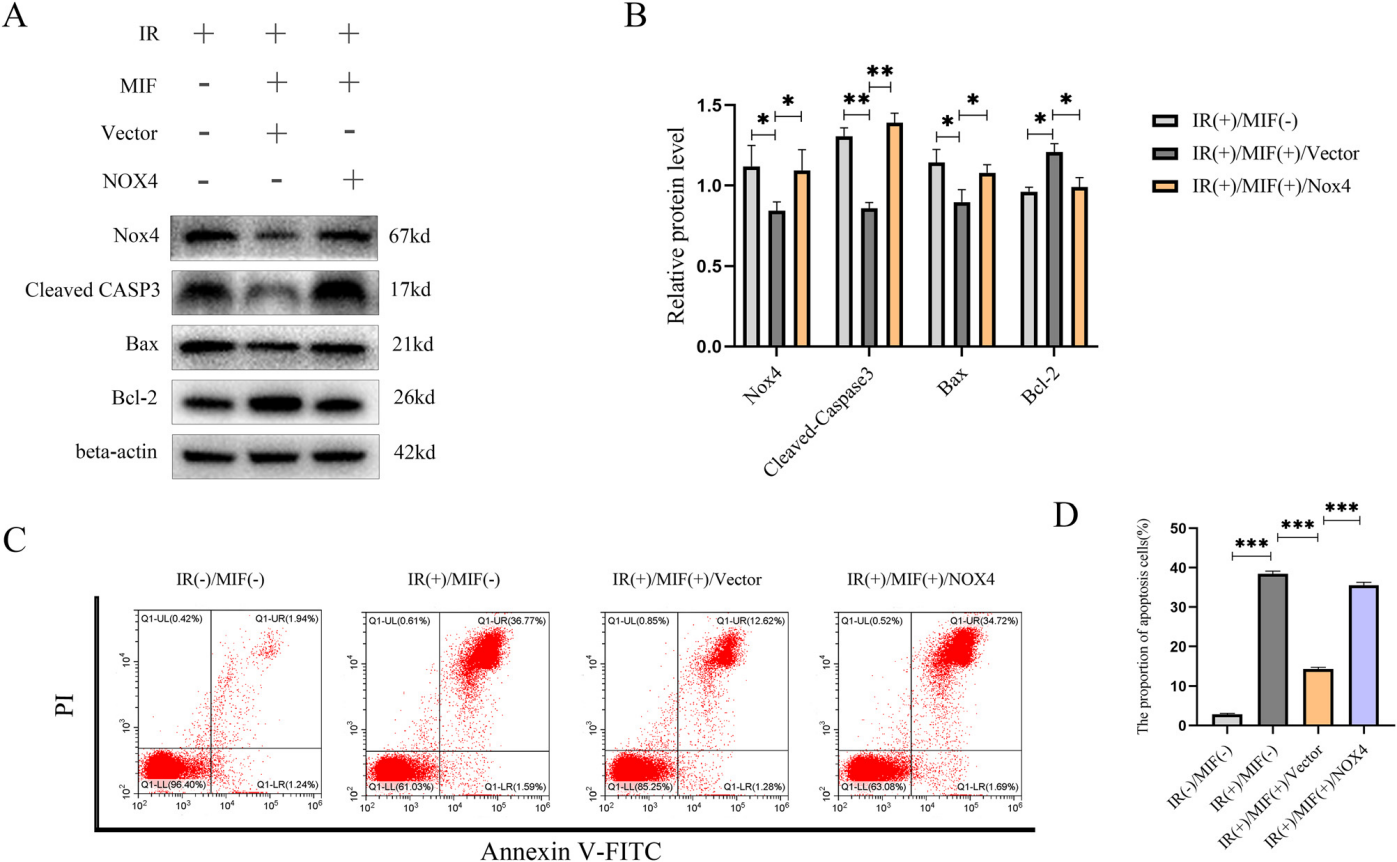
Overexpression of NOX4 signal pathway can inhibit the protective effect of MIF. (A) Western blot was used to detect the effect of MIF co-transfection with overexpressed NOX4 plasmid or empty plasmid on the expression of NOX4 and apoptosis-related proteins in BMSCs treated with IR. (B) Quantification of the data in panel A. (C) Annexin V/PI double staining was used to detect the effect of MIF co-transfection with overexpressed NOX4 plasmid or empty plasmid on apoptosis of BMSCs treated with IR. (D) Quantify the data shown in panel C. The experiment was repeated three times. (n=3, no significance was noted as “ns”, *p<0.05, **p<0.01, ***p<0.001 using the t-test).

To investigate the effect of NOX4 on IR-induced apoptosis in BMSCs, cells were transfected with a NOX4 overexpression plasmid or empty vector, pretreated with MIF (100 ng/mL) for 24 h, and then exposed to IR. Western blot analysis demonstrated that NOX4 overexpression significantly upregulated the expression of pro-apoptotic proteins Bax and cleaved caspase-3 by 20 % (*p<0.05) and 62 % (**p<0.01), respectively, while downregulating the anti-apoptotic protein Bcl-2 by 19 % (*p<0.05), compared to the vector control group. Concurrently, NOX4 expression was upregulated by 30 % (*p<0.05) following plasmid transfection. (Figure 4A and B). Subsequently, flow cytometry analysis revealed that transfection with the NOX4 overexpression plasmid significantly increased the proportion of apoptotic BMSCs to 29.58 %, compared to 13.9 % in the Vector group (***p<0.001). (Figure 4C and D). These findings indicate that NOX4 overexpression counteracts the protective effect of MIF against IR-induced apoptosis in BMSCs.

### MIF decreased the level of BMSCs ROS

**Figure 5:**
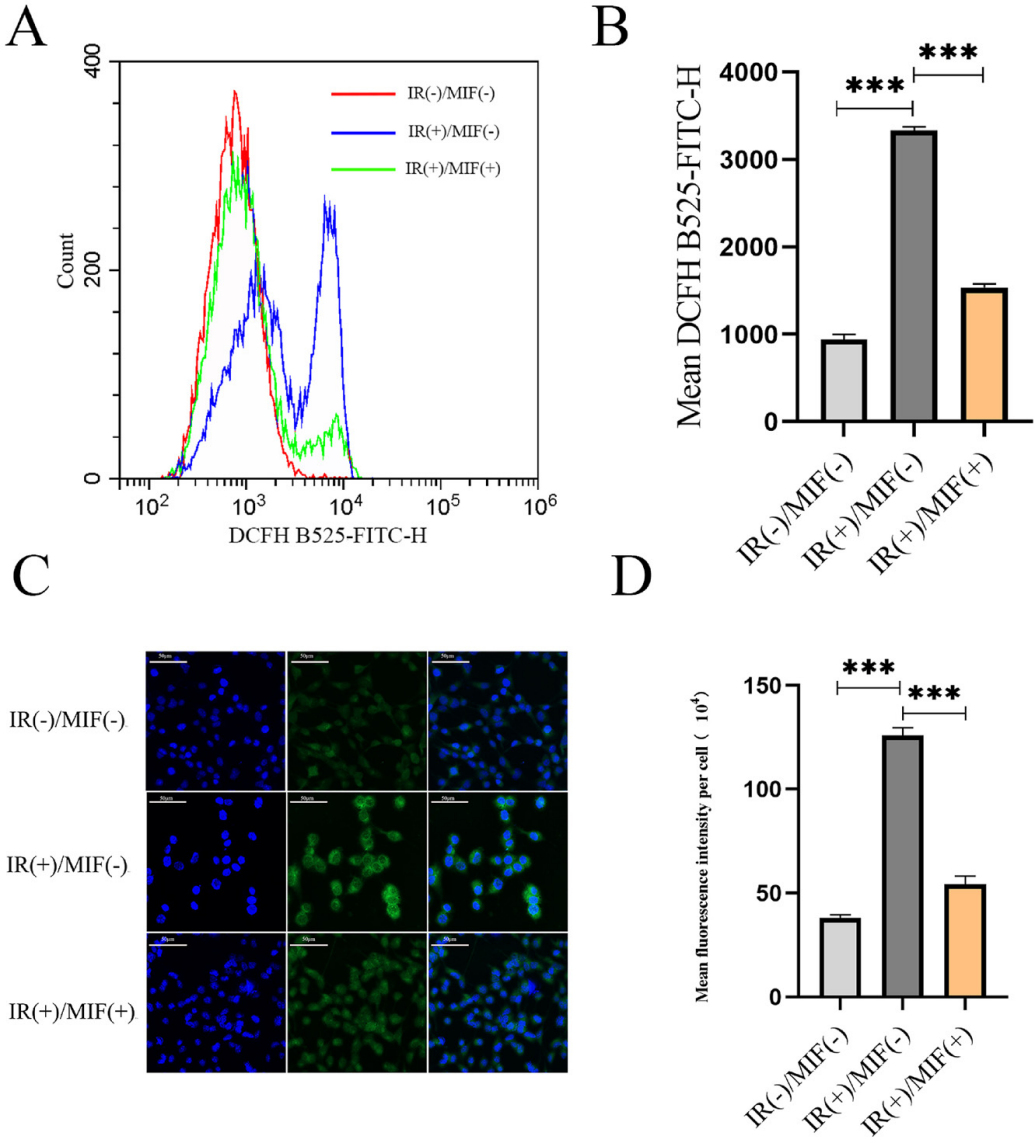
MIF reduces the ROS level of BMSCs. (A) Flow cytometry DCFH staining was used to detect the ROS level of BMSCs in the control group, IR treated group, and MIF treated group before IR treatment. (B) Quantify the data shown in panel A. (C) Fluorescence microscopy images. (D) The mean fluorescence intensity of intracellular ROS levels generated from panel C. (n=3, ***p<0.001 using the t-test).

The results of flow cytometry showed that under IR treatment, the number of BMSCs with high ROS levels increased with the increase in apoptosis rate, while MIF pretreatment significantly decreased the level of ROS (Figure 5A and B). Fluorescence microscopy images showed that the fluorescence intensity of ROS increased after IR treatment, and MIF pretreatment significantly decreased the fluorescence intensity of ROS (Figure 5C and D).

### MIF protects BMSCs from IR-induced apoptosis by downregulating the lncRNA MEG3/NOX4 signaling pathway and reducing ROS levels

**Figure 6:**
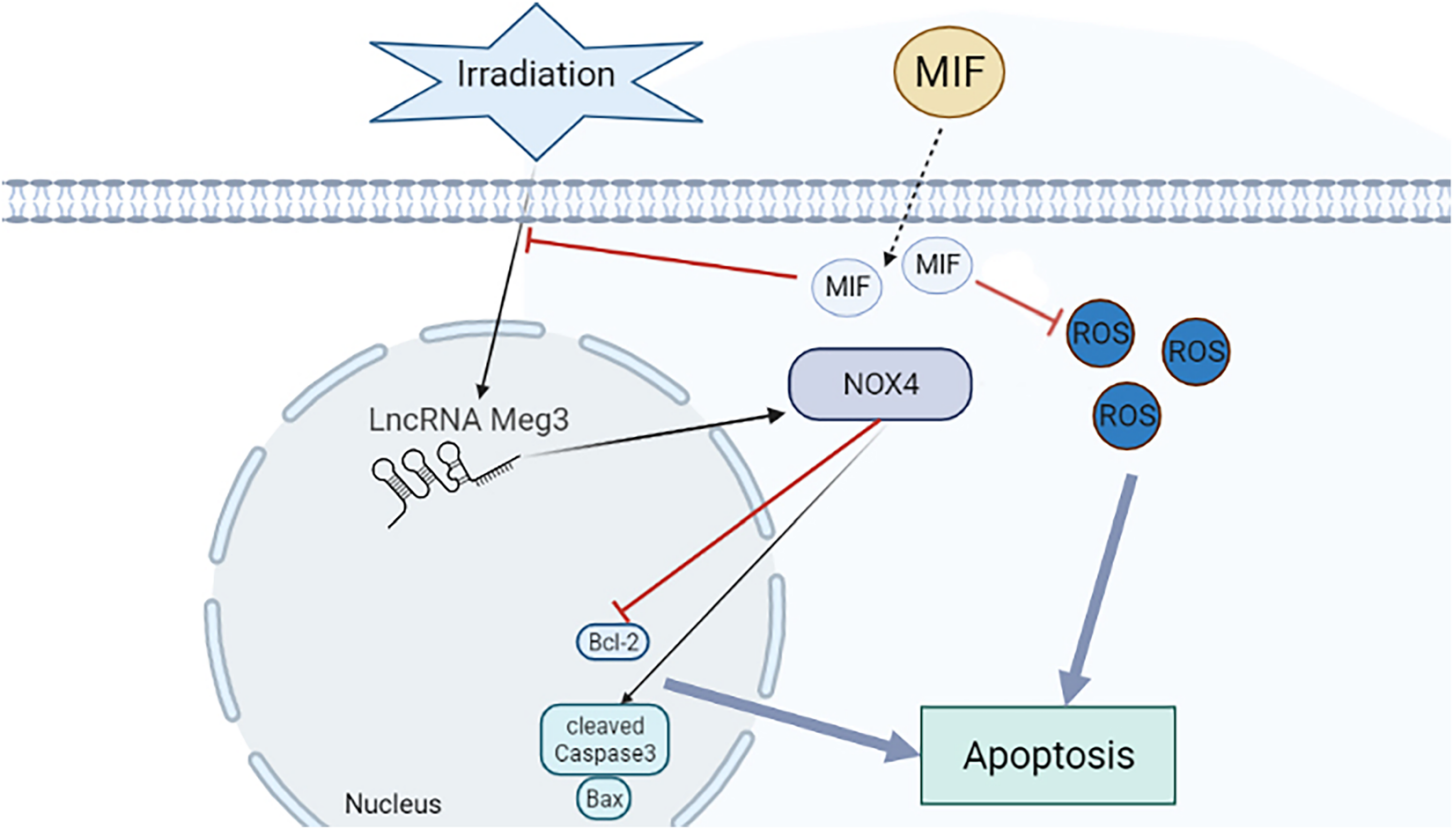
MIF protected BMSCs from IR induced apoptosis by downregulating the lncRNA MEG3/NOX4 signaling pathway and decreasing ROS levels. The yellow arrow represents promoting effect and the red inhibition arrow represents the downregulation. IR stimulation induced apoptosis by promoting MEG3/NOX4 signaling pathway, exogenous MIF reduced ROS levels and also downregulate MEG3/NOX4 alleviated apoptosis.

MIF plays a protective role in mitigating IR-induced apoptosis in BMSCs by targeting the lncRNA MEG3/NOX4 signaling pathway and reducing ROS levels, IR stimulation promotes apoptosis through the activation of the MEG3/NOX4 signaling pathway, while exogenous MIF treatment counteracts this effect by downregulating MEG3/NOX4 expression and reducing ROS levels (Figure 6). These findings demonstrate that MIF protects BMSCs from IR-induced damage by modulating the lncRNA MEG3/NOX4 pathway and oxidative stress.

## Discussion

In the past, the focus of tissue regeneration therapy for RANFH was mainly on osteogenesis and vascularization after transplantation of stem cells [33], but only a small amount of exogenous stem cells remained in the injury site after transplantation, which greatly limits the therapeutic effect. Therefore, how to reduce BMSCs apoptosis and regulate oxidative stress response and maintain the viability of transplanted cells and promote continuous function are the key factors for the success or failure of treatment [34]. In this study, we reveal the mechanism of BMSCs apoptosis in IR induced oxidative stress environment.

Previous studies have confirmed that lncRNA MEG3 is closely related to cellular oxidative stress and DNA damage. In diabetes, lncRNA MEG3 mediates apoptosis of microangiopathy cells leading to significant cellular oxidative stress and DNA damage [35], 36]. It has also been reported that in the model of cerebral ischemia, down-regulation of lncRNA MEG3 can inhibit the occurrence of oxidative stress and inflammation [37]. In this study, the expression of lncRNA MEG3 in IR-treated BMSCs was detected by RT-qPCR, revealing that the high oxidative stress environment induced by IR significantly increases lncRNA MEG3 expression in BMSCs.These results suggest that regulation of lncRNA MEG3 is an effective way to promote BMSCs to resist apoptosis induced by high oxidative stress environment in BMSCs.

MIF is a pluripotent cytokine that plays an important role in the cellular stress response [38]. Previous studies have shown that MIF can provide cardiac protection during ischemia/reperfusion by reducing oxidative stress [39]. In addition, MIF exhibits oxidoreductase activity, participating in the regulation of oxidative cell stress and displaying anti-apoptotic properties in various environments [40]. In previous studies, it was found that MIF had a regulatory effect on lncRNA. Studies showed that the expression of exosomes extracted from MIF-pretreated MSCs was significantly increased compared with that from untreated MSCs, and played a role in myocardial protection [41]. Recent studies have found that MIF can significantly inhibit apoptosis and reduce oxidative stress by inhibiting LncRNA-p21 [24]. Recent studies have shown that anti-TGF-β/PD-L1 bispecific antibody synergizes with radiotherapy to enhance antitumor immunity while mitigating radiation-induced pulmonary fibrosis [42]. This crosstalk between radiotherapy and immunological signals helps to better appreciate MIF’s protective function under IR-induced injury, particularly in the context of immune cell modulation within stress environments, as highlighted by recent work on CCR5+ T cell regulation in the tumor microenvironment [42]. In this study, we confirmed that MIF can protect BMSCs from apoptosis induced by high oxidative stress caused by IR by acting on lncRNA MEG3 and reducing its expression.

Under various stress conditions, including ischemia, hypoxia, and aging, NOX4 has been shown to exert cytoprotective effects through the regulation of oxidative stress [39]. Emerging evidence further indicates a regulatory relationship between lncRNAs and NOX4. For instance, in hepatic sinusoidal endothelial cells, lncRNA H19 contributes to hypoxic stress by positively modulating NOX4 expression [43], while in cancer stem cells, lncRNA MEG3 activates NOX4 to promote apoptosis [44]. Consistent with these findings, our study demonstrates that transfection of a NOX4 overexpression plasmid effectively abolishes the protective effect of MIF against oxidative stress injury in BMSCs. These results suggest that the MEG3/NOX4 axis plays a critical role in mediating cellular responses to stress. Regarding the downstream signaling mechanisms, the MEG3/NOX4 axis may involve the MEK/ERK pathway. This is supported by studies such as one showing that *S. aureus* upregulates MUC13 to modulate mucosal remodeling in chronic rhinosinusitis through MEK1/2 and WNT2B signaling [45], highlighting potential parallels in redox-sensitive remodeling processes. Additionally, a recent study reported that nicotine-induced activation of CHRNA5 modulates CES1 expression and promotes cancer progression via the MEK/ERK pathway [46], further underscoring the involvement of MEK/ERK in oxidative stress responses and corroborating our observations on the MEG3/NOX4 axis. Collectively, our results indicate that MIF enhances the antioxidant capacity of BMSCs and promotes their survival under oxidative stress by suppressing the lncRNA MEG3/NOX4 signaling pathway.

Previous studies have shown a close relationship between oxidative stress and radiation [47], the main characteristic of oxidative stress is the production of ROS [48]. The enhancement of oxidative stress leads to the further accumulation of ROS and other harmful components, which eventually leads to aging cell dysfunction and apoptosis and necrosis [49]. Recent studies have shown that transglutaminase two inhibition ameliorates cardiac fibrosis by inducing M2 macrophage polarization, underscoring the importance of immune modulation in oxidative stress responses [50], this provides valuable context for understanding MIF’s potential role in regulating anti-apoptotic pathways and maintaining redox balance through immunomodulatory mechanisms, further exploration in future studies remains warranted. In this study, BMSCs were treated with IR *in vitro* to simulate oxidative stress, the results suggesting that MIF can decrease ROS level of BMSCs and decrease the apoptosis rate of BMSCs.

In short, MIF may be able to promote the survival of BMSCs under high oxidative stress environment. Our results show that MIF can protect BMSCs from IR-induced apoptosis by down-regulating the lncRNA MEG3/NOX4 signaling pathway and reducing the level of ROS. These results provide a basis for the clinical application of BSMCs transplantation.

## Study limitations

While the current study provides substantial evidence supporting the role of the MIF-mediated lncRNA MEG3/NOX4 pathway in protecting BMSCs from IR-induced apoptosis, certain mechanistic validations – such as genetic knockout models – were not included. These approaches, though highly informative, require extended timelines for the development of stable cell lines or animal models. We will prioritize knockout-related validation work in subsequent research to strengthen the evidence for our conclusions.
